# A deep learning model for automated kidney calculi detection on non-contrast computed tomography scans in dogs

**DOI:** 10.3389/fvets.2023.1236579

**Published:** 2023-09-20

**Authors:** Yewon Ji, Gyeongyeon Hwang, Sang Jun Lee, Kichang Lee, Hakyoung Yoon

**Affiliations:** ^1^Department of Veterinary Medical Imaging, College of Veterinary Medicine, Jeonbuk National University, Iksan, Republic of Korea; ^2^Division of Electronic Engineering, College of Engineering, Jeonbuk National University, Jeonju, Republic of Korea

**Keywords:** artificial intelligence model, renal calculi, urolithiasis, computed tomography, canine

## Abstract

Nephrolithiasis is one of the most common urinary disorders in dogs. Although a majority of kidney calculi are non-obstructive and are likely to be asymptomatic, they can lead to parenchymal loss and obstruction as they progress. Thus, early diagnosis of kidney calculi is important for patient monitoring and better prognosis. However, detecting kidney calculi and monitoring changes in the sizes of the calculi from computed tomography (CT) images is time-consuming for clinicians. This study, in a first of its kind, aims to develop a deep learning model for automatic kidney calculi detection using pre-contrast CT images of dogs. A total of 34,655 transverseimage slices obtained from 76 dogs with kidney calculi were used to develop the deep learning model. Because of the differences in kidney location and calculi sizes in dogs compared to humans, several processing methods were used. The first stage of the models, based on the Attention U-Net (AttUNet), was designed to detect the kidney for the coarse feature map. Five different models–AttUNet, UTNet, TransUNet, SwinUNet, and RBCANet–were used in the second stage to detect the calculi in the kidneys, and the performance of the models was evaluated. Compared with a previously developed model, all the models developed in this study yielded better dice similarity coefficients (DSCs) for the automatic segmentation of the kidney. To detect kidney calculi, RBCANet and SwinUNet yielded the best DSC, which was 0.74. In conclusion, the deep learning model developed in this study can be useful for the automated detection of kidney calculi.

## 1. Introduction

Urinary calculi in the kidneys and upper and lower urinary tracts are among the most common abnormal findings in canine urinary disorders. According to a recent study, prevalence of upper urinary tract and lower urinary tract uroliths were reported to be 19 and 41%, respectively (1). In dogs, most urinary calculi are reported to be in the lower urinary tract, for example, the bladder and urethra, or are voided in the urine (2). Less than 3–4% of all urinary calculi in dogs are located in the renal pelvis (2, 3), while most human patients with urinary calculi are reported to have nephroliths (3).

Renal calculi can be asymptomatic in many dogs; however, when the size or location of the calculi change, they are no longer silent, and can lead to clinical problems such as partial or complete ureteropelvic junction obstruction, hydronephrosis, renal parenchymal loss due to growing calculi, hematuria, and urinary tract infection due to infected calculi (4). In addition, a study in human medicine has also reported that the renal calculi can be associated with the increasing risk of chronic kidney diseases (5–8).

Therefore, early detection and size quantification of urinary calculi are important to prevent severe kidney diseases associated with calculi, and to provide better and timely treatment. Owing to their importance, several diagnostic imaging modalities, including X-rays, ultrasound, and computed tomography (CT), have been used to detect urinary calculi in both veterinary and human medicine. Among these methods, CT is reported to be the most accurate for detecting calculi with high sensitivity and specificity (9). However, the limitation of these methods lies in the time-consuming nature of evaluation and size quantification of renal calculi in clinical field, as it is performed by manually measuring the size and the number of calculi.

Of late, numerous studies in human medicine have shown that deep learning models can be successfully applied to medical imaging fields for aspects such as classification, segmentation, and lesion detection (10–13). Convolutional neural networks, a recent advancement in deep learning-based analysis methods, have shown promising performance in these tasks (14). To date, several novel architectures have been proposed for training using medical images. Attention U-Net (AttUNet), which integrates an attention gate into the U-Net model, consistently improves the prediction performance of U-Net on abdominal CT datasets for multiclass image segmentation (15). Recently, a hybrid transformer architecture called UTNet was proposed. UTNet integrates self-attention into a convolutional neural network that allows the initialization of transformer models without the need for a pre-training weight, while transformers require a large amount of data to learn vision inductive bias (16). In addition, TransUNet, an architecture using Transformer as an encoder in combination with U-Net aims to enhance the finer details, and has yielded promising performance on medical images for multi-organ segmentation and cardiac segmentation (17).

In human medicine, many studies have proposed various deep learning models for the segmentation and detection of kidneys (18–20) and kidney tumors (21–23). Many recent human medicine studies have proposed deep learning models with several architectures for automatic kidney stone detection on CT images (24–27). In veterinary medicine, a recent study proposed a deep learning model based on the UNet Transformer to detect the kidney and automatically estimate its volume from the pre- and post-contrast CT images of dogs (28). However, no deep learning model has yet been proposed for the automated detection of kidney calculi from CT images in veterinary medicine.

In this study, we aimed to develop deep learning models for the automatic detection of kidney calculi and kidneys from non-contrast CT scans in dogs, and to evaluate the performance of these models.

## 2. Materials and methods

### 2.1. Dataset for CT scans

A total of 167 pre-contrast CT scans (instruments used were as follows: Alexion, TSX-034A, Canon Medical System Europe B.V. and Zoetermeer, Netherlands; Revolution ACT, GE Healthcare, Milwaukee, WI, USA; and Brivo CT385, GE Healthcare, Milwaukee, WI, USA) of 167 dogs were randomly collected from multiple centers. Among the 167 pre-contrast CT scans, 34,655 transverseimages from 76 CT scans included kidney calculi, and were used for training and validation. The imaging protocols were as follows; 120 kVp, 150 mAs, 512 × 512 matrix and 0.75 rotation time (Alexion); 120 kVp, 84 mAs, 512 × 512 matrix, and 1 rotation time (Revolution ACT); and 120 kVp, 69 mAs, 512 × 512 matrix, and 1 rotation time (Brivo CT385). The slice thickness of the CT scans included in the study varied from 0.75 mm to 2.5 mm. Postcontrast CT scans were not included in the present study.

The precontrast CT images included in this study were divided into training and validation data at a ratio of 80 to 20. Therefore, a total of 61 CT scans were randomly chosen as the training data and 15 CT scans were used as the validation data. The CT scans of dogs without medical records were excluded from the study. In addition, scans with motion artifacts, without volume information, or with an axis smaller than a certain size were excluded.

### 2.2. Patient dataset

In this study, 76 CT scans from 76 dogs with kidney calculi who underwent CT scans were included. The medical records of the dogs, including data on age, sex, neutering status, body weight, and laboratory examination results, were collected.

### 2.3. Manual segmentation

**Figure 1:**
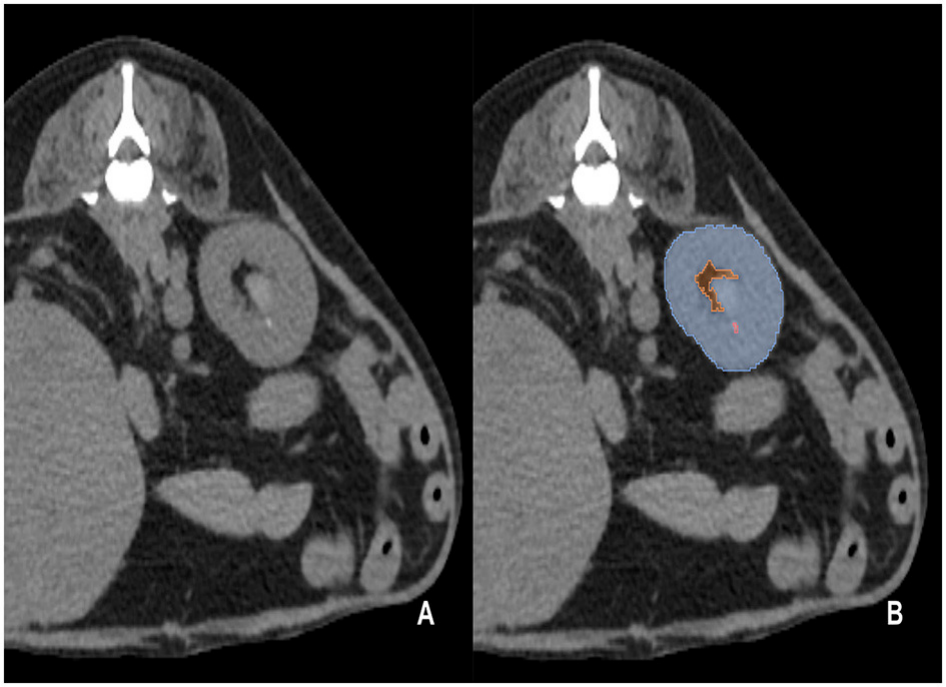
Examples of manual segmentation; original CT images at the level of kidney **(A)** and the example of manual segmentation **(B)**. The kidneys in the pre-contrast images were manually segmented using a segmentation tool (Medilabel software). In the pre-contrast images, the kidneys were segmented into three classes: parenchyma (Class 1, light blue color in the labeled image), renal pelvis, and surrounding fat (Class 2, orange color in the labeled image). Calculi in the kidneys were segmented into Class 3 (light pink color in the labeled image).

The pre-contrast CT scans of the dogs included in the study were manually segmented by 10 clinicians (residents at the Veterinary Medical Imaging Department of the Teaching Hospital of Jeonbuk National University) using the Medilabel software (Ingradient, Inc., Seoul, South Korea). From the images, all kidneys were segmented into the following three classes: (1) renal parenchyma; (2) renal pelvis and surrounding fat; and (3) calculi (Figure 1). The renal pelvis and the fat around it were labeled separately to prevent false training results wherein the model recognizes the fat around the pelvis as the kidney.

### 2.4. Pre-processing

The training data were converted into a numpy array and pre-processed using the following steps: data resampling, intensity normalization, and non-zero region cropping, using Python and Pytorch framework.

A non-zero crop is a process used for exclusively obtaining the actual region of interest (RoI) by cropping out the background. External structures, such as fixation frames for fixing animals on the CT table during scans, were masked, and voxels with certain values (under−1000 Hounsfield Unit) were considered as the background, and cropped. Data were resized to 512 × 512 pixels.

Intensity normalization was performed to clip the minimum and maximum to Hounsfield Unit (HU) values of −155 and 195, respectively.

To address variations in the spatial spacing and slice thickness of the CT scans used in this study, resampling was performed to adjust the various pixel dimensions and standardize the data to an isotropic voxel spacing of x = 0.5, y = 0.5, and z = 1.4 (mm). To preprocess the training data, which only included pre-contrast CT scans, the window width and level were set to 350 and 30 HU, respectively, and the minimum and maximum HUvalues were clipped to −155 and 195, respectively, before applying min-max normalization (Minimum = Window level - $\frac{Window\;width}{2}$, Maximum = Window level + $\frac{Windowwidth}{2}$).

This technique ensured that the intensity values of the images were consistent and comparable across different scans.

### 2.5. Model architecture

**Figure 2:**
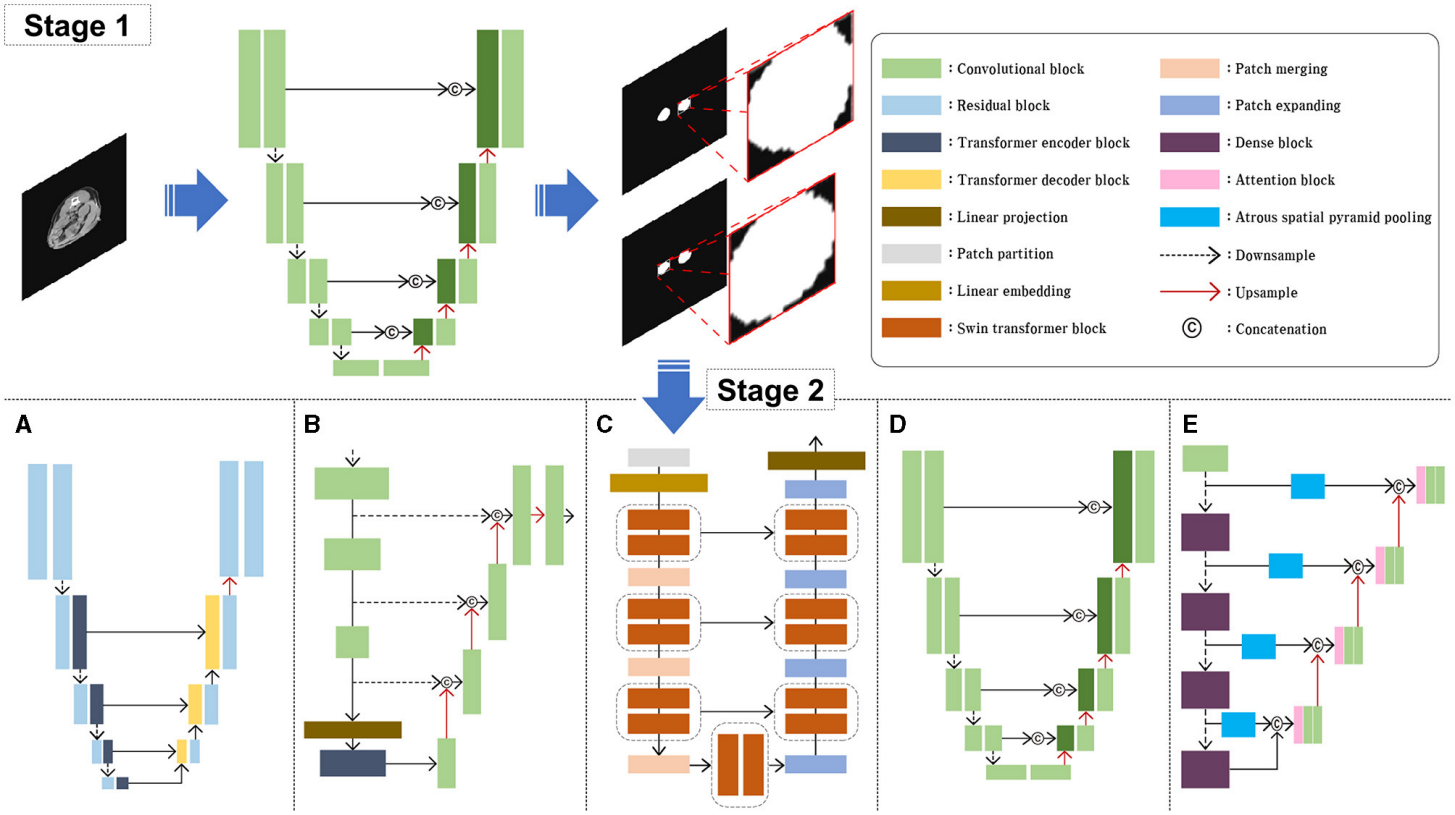
Schematic illustration of the model architectures used in this study. For the stage 1, AttUNet, a convolution-based model integrated with attention gate was used. For the stage 2, five different models were used and compared. UTNet **(A)**, TransUNet **(B)**, nd SwinUNet **(C)** are architectures based on transformer models. AttUNet **(D)** and RBCANet **(E)** are based on convolution models.

In this study, several model architectures previously employed for various image segmentation tasks were utilized, including Attention U-Net (AttUNet) (15), UTNet (16), TransUNet (17), SwinUNet (29), and RBCANet (21); these have previously been used for various image segmentation tasks. The overall block diagram of the model architecture is shown in Figure 2. Five different models based on transformer and CNN were selected based on the reliability and efficiency from previous studies which showed high accuracy and stability on medical images such as CT and Magnetic Resonance Imaging (MRI) in human medicine.

The AttUNet model extends the original U-Net architecture by incorporating attention gates into the skip connections (15). The architecture maintains the encoder-decoder structure of the U-Net, with downsampling layers in the encoder and upsampling layers in the decoder. Attention gates are added to the skip connections, allowing the model to focus selectively on the most relevant features in the input image. The attention gates learn spatial dependencies and feature importance using attention mechanisms that are often implemented through additive or multiplicative approaches. This selective focus on relevant features leads to improved segmentation performance.

UTNet has a U-shaped architecture, similar to the original U-Net, with an encoder-decoder structure and skip connections (16). The main difference lies in the encoder part, which consists of transformer-based layers instead of standard convolutional layers. These layers capture both local and global features from the input image, while the decoder uses convolutional layers to upsample the feature maps and generate the final segmentation map. The combination of transformers and convolutions allows UTNet to effectively segment images with complex structures, such as ultrathin endoscope images.

TransUNet combines the U-Net architecture with a vision transformer to create a hybrid model (17). The vision transformer is used as an encoder, replacing the standard convolutional layers of the U-Net architecture. The vision transformer divides the input image into non-overlapping patches and processes them using self-attention and positional encoding, allowing it to effectively capture global contextual information. The decoder part of TransUNet remains similar to that of the original U-Net, using upsampling layers and skip connections to generate the final segmentation map. This combination of the vision transformer and U-Net architecture enables TransUNet to capture both local and global context information, resulting in improved segmentation performance.

SwinUNet incorporates the Swin transformer as its encoder, replacing the standard convolutional layers of the U-Net architecture (29). The swin transformer is a hierarchical transformer-based architecture that uses shifted windows to process input images, capturing both local and global context information while maintaining relatively low computational complexity. The decoder part of SwinUNet retains the original U-Net design, and consists of upsampling layers and skip connections. By combining the strengths of both the swin transformer and the U-Net architecture, SwinUNet achieves improved performance in various image segmentation tasks.

The RBCANet architecture utilizes a pre-trained DenseNet-161 encoder and U-Net's encoder–decoder structure to effectively capture hierarchical features (21). The atrous spatial pyramid pooling module, integrated within the skip connections, processes input features at various scales, capturing both local and global contextual information that is crucial for accurate segmentation. Working in conjunction with the Reverse Boundary Attention and Channel Attention modules, RBCANet improves the segmentation performance by emphasizing accurate boundary predictions and focusing on the most informative channels.

One of the main impediment factors of this study was the variation of data source owing to the relatively small size of calculi in the full CT images, various size of dogs and the fact that the data included in this study were collected from multicenter. To overcome this problem, our approach involved two stages and employed five architectures. In Stage 1, we utilized an AttUNet-based model to obtain an approximate RoI of the kidney through coarse feature maps. This step involved automatic segmentation of the kidney from the input CT image, followed by extraction of the RoI by cropping a non-zero region that excludes the kidney. The extracted RoI was then resized to 128 × 128 pixels and used as input for Stage 2.

In Stage 2, the RoI obtained from Stage 1 was used as input. We evaluated the performance of five models for kidney stone segmentation: UTNet (Figure 2A), TransUNet (Figure 2B), SwinUNet (Figure 2C), Attention U-Net (Figure 2D), and RBCANet (Figure 2E). The performance of each model was compared across different architectures to determine the most effective approach for kidney stone segmentation in CT images.

### 2.6. Implementation details

The input channel 1 and the output channel 3 were utilized. The PyTorch framework was used to construct the models. The combined loss function, including the weight addition of the cross-entropy and the dice loss function, is known to improve the performance of the segmentation network (30–33). In this study, a combined loss function including generalized dice loss and focal loss was used to improve the performance of the models (even with data imbalance). The loss function used in this study was as follows:


$$\begin{array}{l} Loss\;function=\alpha *Generalized\;DiceLoss\\ \;+\beta *FocalLoss(\alpha =1,\;\beta =0.5) \end{array}$$

$$\begin{array}{l} Generalized\;Dice\;Loss=1-2\frac{\sum_{l=1}^{2}w_{l}\sum_{n}r_{\ln}p_{\ln}}{\sum_{l=1}^{2}w_{l}\sum_{n}r_{\ln}+p_{\ln}} \end{array}$$

(*p*_*n*_, predicted map of foreground label of number of image elements; *r*_*n*_, ground truth of kidney and calculi; *l*, foreground label; *w*_*l*_, ${\left(\frac{1}{\overset{N}{\underset{n=1}{\sum }}r_{\ln}}\right)}^{2}$, weight addition for the number of label pixels)


$$Focal\;loss=-{\left(1-p_{t}\right)}^{\gamma }\log(p_{t})\;(p_{t}=\{{}_{1-p\;otherwise}^{p\;if\;y=1},\;\gamma =2)$$

To improve the model training performance, data augmentation was performed using ShiftScaleRotate, GridDistortion, Opticaldistortion, ElasticTransform, CoarseDropout, and GaussNoise from the albumentations library (34). The parameters for each step were as follows: scale limit (−0.2, 0.2) and rotate limit (−180, 180) were used for ShiftScaleRotate; the number of grid cells was five for each side, and the distort limit was set to (−0.03, 0.03); the distort limit for the optical distortion was set to (−0.05, 0.05); ElasticTransform was performed by displacement fields to convert pixels, and α and σ were set to 1.1 and 0.5, respectively; the maximum height and minimum width were set to 8 for CoarseDropout; and GaussNoise was assigned a value of gaussian noise (0, 0.001) and an average of 0. In this study, data augmentation was applied only to the training process and not to the validation process.

Deep learning model training was conducted for 100 epochs using an NVIDIA RTX 3090, Python, and PyTorch framework graphics processing unit. For the training, exponentially learning rate scheduler was applied and each random data augmentation was performed with a probability of 0.5, and a learning rate of 0.01. SGD optimizer was used for the training with a batch size of 32, momentum of 0.9, and weight decay of 1e-4.

### 2.7. Model metrics and statistical evaluation

Several evaluation metrics were used to evaluate model performance. Dice Similarity coefficient (DSC) measures the relative voxel overlap between the ground truth and the predicted segmentation to evaluate the similarity between segmentations using an automated model and the ground truth. A DSC close to one implies high similarity. The DSC was measured using the following formula: $DSC=\frac{2|S_{g}\cap S_{p}|}{\left|S_{g}|+|S_{p}\right|}$ (*S*_*p*_, predicted pixel value; *S*_*g*_, segmentation pixel value of ground truth).

Intersection over Union, which is similar to DSC but penalizes under-segmentation and over-segmentation more than the DSC, was also used. The formula for Intersection over Union is as follows: $\frac{\left|S_{g}\cap S_{p}\right|}{\left|{S{}_{g}}_{p}\right|}\;$(*S*_*p*_, predicted pixel value; *S*_*g*_, segmentation pixel value of ground truth).

As sensitivity and specificity are recognized as standard metrics for performance evaluation in the medical field, both of the above were used in this study (35, 36). They were calculated as below:

Sensitivity = $\frac{TP}{TP+FN}$ (TP, true positive; FN, false negative); Specificity = $\frac{TN}{TN+FP}\;$(TN, true negative; FP, false positive).

Precision and Accuracy were also used to evaluate the models. The formulae for precision and accuracy are as follows: Precision = $\frac{TP}{TP+FP}$ (TP, true positive; FP, false positive); Accuracy = $\frac{TP+TN}{TP+TN+FP+FN}$ (TP, true positive; TN, true negative; FP, false positive; FN, false negative).

Receiver Operating Characteristic (ROC) is a line plot that depicts the diagnostic ability of a classifier based on its performance with different thresholds. A ROC curve was established as a standard metric for comparing multiple models, and was used to evaluate the models (35). The area under the curve (AUC) shows the performance of models across different thresholds and provide an aggregate measure range from 0 to 1. The result near 1 implies higher performance.

For the statistical evaluation of the characteristics of the dogs included in the study, Kolmogorov–Smirnov test and Shapiro–Wilk test were performed as tests for normal distribution. Mann–Whitney tests were used to evaluate differences in age among dogs with and without kidney calculi.

## 3. Results

### 3.1. Evaluation of five models for kidney detection on pre-contrast CT scans

In Stage 1, SwinUNet showed the best DSC (0.943), followed by RBCANet (0.942), UTNet (0.935), and AttUNet and TransUNet (0.934). As the DSC measures the relative pixel overlap between the manual segmentation and the prediction of the models, DSC close to 1 is considered to have higher similarity between two segmentations in this study.

The sensitivity and specificity at the Youden point of the models were the highest for RBCANet (sensitivity 0.96, specificity 0.95), followed by TransUNet (sensitivity 0.95, specificity 0.94), SwinUNet (sensitivity 0.95, specificity 0.96), UTNet (sensitivity 0.95, specificity 0.95), and AttUNet (sensitivity 0.94, specificity 0.95).

**Figure 3:**
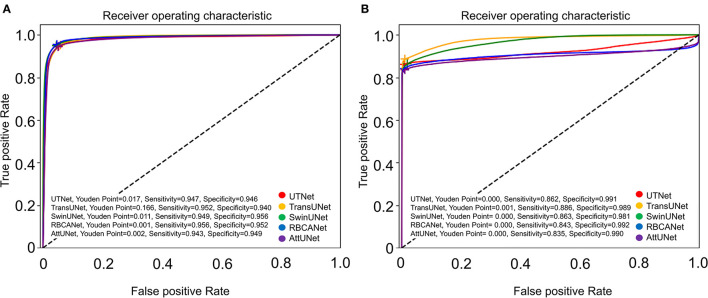
Receiver Operator Characteristic (ROC) curves of the five models. **(A)** Shows the ROC curves of the models for automatic segmentation of the kidneys. Area-under-the-curve (AUC) values for the models are as follows: 0.99 (TransUNet, SwinUNet), and 0.98 (UTNet, RBCANet, AttUNet). **(B)** Shows the ROC curves of the models for the automatic detection of kidney calculi. The AUC values of the models are as follows: 0.98 (TransUNet), 0.97 (SwinUNet), 0.92 (UTNet), 0.91 (RBCANet), and 0.90 (AttUNet). The sensitivity and specificity at the Youden Point are shown in the graphs.

The ROC curve for kidney detection for the five models on the test set is shown in Figure 3A. The AUCfor detecting the kidney was 0.99 for TransUNet and SwinUNet, and 0.98 for UTNet, RBCANet, and AttUNet. The AUC close to 1 is considered to have better performance in this study.

The IOU and precision metrics of the models are summarized in Table 1.

**Table 1:** Quantitative results of the models for detecting kidneys.

	**UTNet**	**TransUNet**	**SwinUNet**	**AttUNet**	**RBCANet**
DSC	0.93446	0.93393	0.94318	0.93418	0.94176
IoU	0.87699	0.87605	0.89248	0.87649	0.88993
Sensitivity	0.92480	0.94072	0.93632	0.92298	0.92660
Specificity	0.96267	0.94944	0.96632	0.96368	0.97179
Precision	0.93985	0.91745	0.93958	0.93656	0.95007
Accuracy	0.95432	0.9506	0.95889	0.95583	0.95979

DSC, Dice Similarity Coefficient; IoU, Intersection over Union; Sensitivity and Specificity refers to that at the Youden point.

### 3.2. Evaluation and comparative analysis of the models for kidney calculi detection on pre-contrast CT scans

The performance of several models in detecting kidney calculi was assessed in this study. AttUNet outperformed the other models, with the highest DSC of 0.741, suggesting its superior capability to accurately delineate the intricate structures of kidney calculi. The DSCs of the other models were as follows: SwinUNet, 0.736; RBCANet, 0.733; UTNet, 0.701; and TransUNet, 0.682.

In terms of sensitivity and specificity at the Youden point, TransUNet topped the list with values of 0.89 and 0.99, respectively. UTNet, SwinUNet, RBCANet, and AttUNet also exhibited competitive sensitivity and specificity values, demonstrating their ability to correctly identify true positive and true negative cases. The sensitivity and specificity of the models are summarized in Table 2.

**Table 2:** Quantitative results of the models for detecting kidney calculi.

	**UTNet**	**TransUNet**	**SwinUNet**	**AttUNet**	**RBCANet**
DSC	0.70051	0.68243	0.73590	0.74108	0.73277
IoU	0.53907	0.51795	0.58215	0.58867	0.57825
Sensitivity	0.86189	0.88586	0.86326	0.84275	0.83546
Specificity	0.99078	0.98882	0.98086	0.99151	0.99015
Precision	0.66803	0.61981	0.73345	0.72945	0.72854
Accuracy	0.99876	0.99861	0.99896	0.99894	0.99896

DSC, Dice Similarity Coefficient; IoU, Intersection over Union; Sensitivity and Specificity refers to that the Youden point.

**Figure 4:**
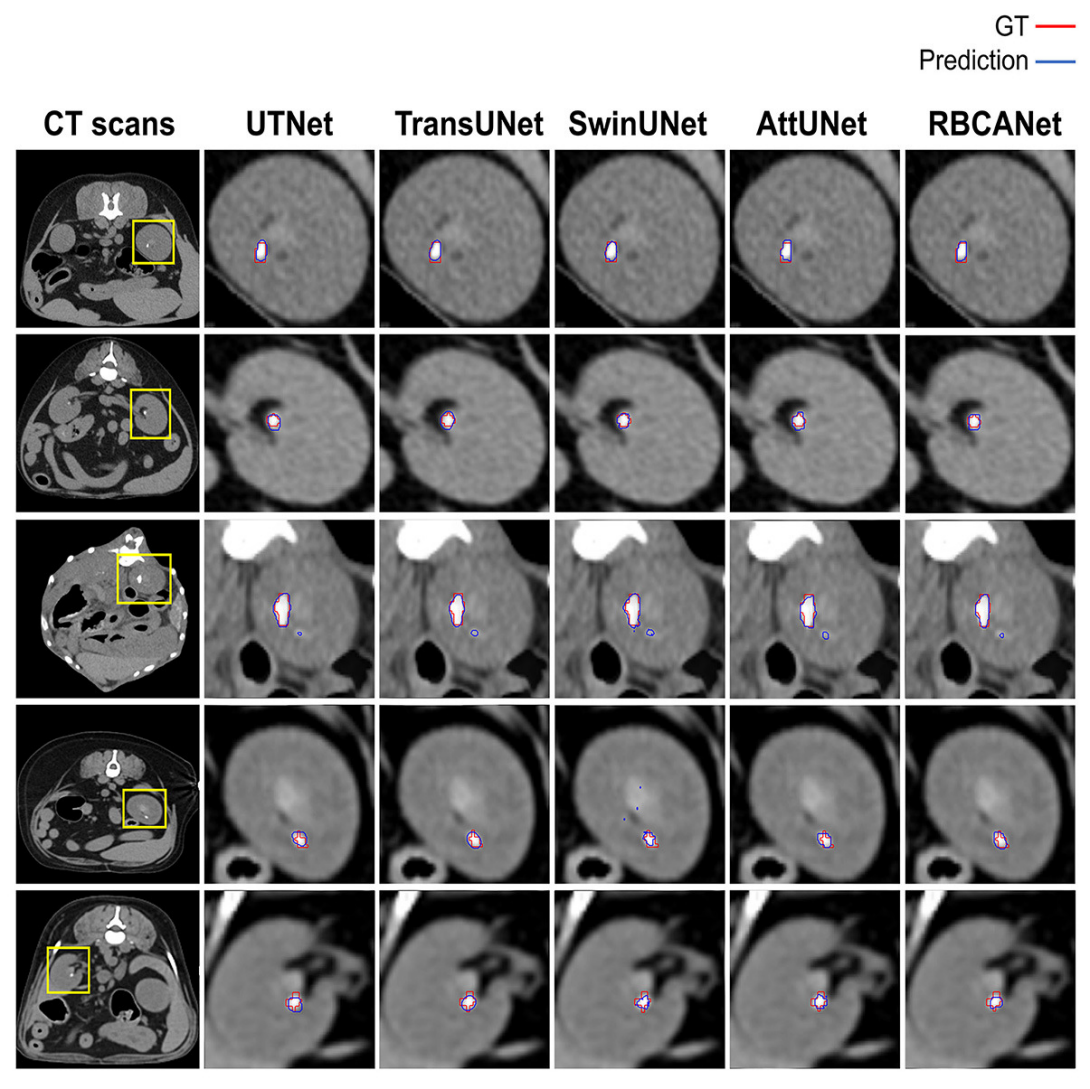
Manual visual analysis of segmented kidneys and kidney calculi (Ground Truth, Red) and the predictions (Predictions, Blue) generated by the models dependently.

Further comparisons were made based on the ROC curves shown in Figure 3B. TransUNet yielded the highest AUC of 0.98 for calculi detection, indicating its superior ability to differentiate between positive and negative cases of kidney calculi. The AUCs for the other models (in decreasing order) were as follows: SwinUNet, 0.97; UTNet, 0.92; RBCANet, 0.91; and AttUNet, 0.90. Figure 4 shows the predictive performance of the models with reference to absenteeism, and provides a comparative perspective on their robustness and reliability across different predictive tasks.

### 3.3. Statistical evaluation of dogs included in the study

Among the dogs with kidney calculi included in this study, 32 were neutered males, 3 were intact males, 32 were neutered females, and 9 were intact female dogs. The mean age of dogs with kidney calculi was 10.79 ± 4.00 years (mean ± SD), ranging from 7 months to 18 years. The mean body weight (BW) of the dogs was 5.25 ± 2.49 kg (mean ± SD), ranging from 1.7 kg to 14.85 kg. The distribution of breeds among these dogs was as follow: 21 Malteses, 10 Poodles, 7 Shih Tzus, 6 Yorkshire terriers, 6 mixed breeds, 5 Pomeranians, 5 Schnauzers, 2 Cocker Spaniels, 2 Dachshunds, and 12 others.

Among the dogs without kidney calculi, 45 were neutered males, 6 were intact males, 32 were neutered females, and 8 were intact female dogs. The mean age of the dogs without kidney calculi was 7.72 ± 4.08 years (mean ± SD), ranging from 4 months to 16 years. The mean BW of the dogs was 7.87 ± 7.52 kg (mean ± SD), ranging from 1.75 kg to 41.5 kg. The breed distribution of these dogs was as follows: 18 Malteses, 15 Poodles, 7 Mixed breeds, 7 Shih Tzus, 6 Pomeranians, 4 Cocker Spaniels, 4 Dachshunds, 3 Bichon Frises, and 27 others.

**Figure 5:**
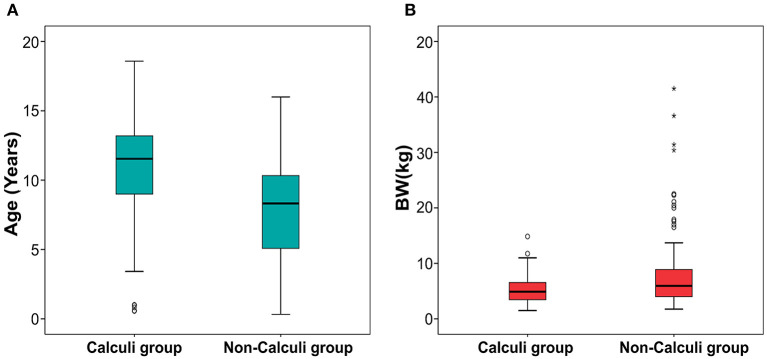
Boxplot of age **(A)** and body weight **(B)** in dogs with and without calculi. Statistically significant difference was found between the dogs with and without calculi for both the parameters. The dogs with calculi were significantly older (*p* < 0.001) and smaller (*p* = 0.005) than the dogs without calculi. The upper and lower edges of the box represent the 25th (Lower quartile, Q1) and 75th (Upper quartile, Q3) percentiles. The vertical line (whiskers) between the lower and upper extremes on each box represents the distribution range of the data. The mild outliers (empty circles) are data points located outside of the whiskers, below Q1 – 1.5 × Interquartile range (IQR) or above Q3 + 1.5 × IQR. The extreme outliers (asterisks) are data points more extreme than Q1 – 3 × IQR or Q3 + 3 × IQR.

Mann-Whitney tests for age (*p* < 0.001) and BW (*p* = 0.005) showed statistically significant differences between dogs with and without kidney calculi. Dogs with kidney calculi were significantly older and smaller than those without calculi. The age and BW of each group are depicted using a box plot in Figure 5.

## 4. Discussion

This is the first study in veterinary medicine to propose deep learning models to detect kidney calculi on CT images of dogs, and to evaluate their performance. All five models developed in this study showed improved performance on detecting kidney from pre-contrast CT images compared to the previous study using UNETR (28). For the kidney calculi detection, the models developed in this study showed promising performance comparable to previous models developed in human medicine.

In this study, detection of the kidney was considered essential for the proper detection of kidney calculi; it was expected that better performance in detecting the kidney would result in a more accurate detection of kidney calculi. Therefore, we developed models that could detect kidney calculi as well as the kidney itself in the first stage of the analysis. Recently, several studies have proposed deep learning models for the automatic segmentation of kidneys on CT images in human medicine. da Cruz et al. (19) reported a model with a DSC of 0.96. Another study reported a model with a DSC of 0.95 and 0.93 for the left and right kidneys, respectively, using ConvNet-Coarse, and 0.94 and 0.93 for the left and right kidney, respectively, using ConvNet-Fine (20). For automatic kidney detection, a previous study in veterinary medicine based on UNet Transformer showed a DSC of 0.912 and 0.915 before and after post-processing, respectively (28). All the models developed in this study showed improved performance for automatic kidney detection compared to this previous study, but showed a slightly lower DSC compared to the models developed for application in human medicine. SwinUNet exhibited the best DSC (0.943), followed by RBCANet (0.942), UTNet (0.935), and AttUNet and TransUNet (0.934). Further studies with more training data and novel architectures can help develop models with DSCs comparable to the models developed in the human medicine field.

In addition, several studies have proposed deep learning models for detecting kidney calculi on CT images in human medicine. Elton et al. (24) reported a sensitivity of 0.88 and specificity of 0.91 on a validation set; Parakh et al. (37) reported a sensitivity of 0.94 and specificity of 0.96 by GrayNet, and sensitivity of 0.90 and specificity of 0.92 by ImageNet. Li et al. (21) evaluated the performances of five different models, and reported that Res U-Net showed a sensitivity of 0.79 and specificity of 0.99, and 3D U-Net showed a sensitivity of 0.80 and specificity of 0.99. The models developed in this study showed a comparable performance to those developed in the field of human medical imaging, with the highest sensitivity value at 0.89 and specificity at 0.99 for TransUNet. Elton et al. (24) reported an AUC of 0.95 on a validation set, while Parakh et al. (37) reported an AUC of 0.954 by GrayNet, and 0.936 by ImageNet on urinary stone detection. In this study, the models using TransUNet, SwinUNet, UTNet, RBCANet, and AttUNet achieved AUCs of 0.98, 0.97, 0.92, 0.91, and 0.90, respectively. TransUNet and SwinUNet performed better than previous models applied in human medicine. Therefore, the use of TransUNet- and SwinUNet-based models for the detection of kidney calculi is promising.

The evaluation of each model in this study reveals its unique strengths and weaknesses. Despite the lower DSC of SwinUNet for the detection of calculi, its high AUC underscores its overall commendable performance. In contrast, UTNet, despite its notable sensitivity and specificity, showed lower DSC and AUC values for calculi detection, suggesting possible difficulties in detecting intricate structures such as kidney stones. Interestingly, AttUNet, despite having the highest DSC (which is indicative of a strong ability to identify kidney calculi), had the lowest AUC, suggesting potential limitations in its overall prediction accuracy. TransUNet demonstrated a balanced performance with the highest AUC but the lowest DSC for kidney calculi detection, suggesting possible challenges for accurate structure delineation. In addition, despite excelling in kidney detection, RBCANet showed a lower DSC and AUC for calculi detection. This suggests that while RBCANet is proficient at handling larger structures (such as kidneys) owing to its effective hierarchical feature capture, it may not be able to effectively identify smaller structures such as kidney stones. Therefore, the selection of an appropriate model should be tailored to the specific task, and should consider the unique strengths and weaknesses of each model.

Compared with the results of models developed in the human medical field, the models developed in this study showed lower DSC values for detecting kidneys. Compared to the current study, a previous study in veterinary medicine using UNet Transformer reported a lower DSC value; a wide range of body sizes associated with various breeds was considered an obstacle in the training process and a factor leading to the lower DSC compared to other models developed for humans (19–21, 28). This is consistent with the observations in the current study.

The main reason for the lower DSC values for detecting kidney calculi of our model compared to those developed in human medicine was the smaller size of the kidneys and calculi. The size of the calculi is reportedly associated with model performance. In a previous study in human medicine, kidney calculi were classified into small (0–6 mm), medium (6–20 mm), and large (above 20 mm) sizes (26). The DSC was highest in large size group, at 83.39 ± 2.33; it was 76.08 ± 3.46 in the middle size group, and the lowest in the small size group, at 60.11 ± 0.84. Among the architectures studied in this previous study, SegNet showed the highest difference in DSC between the large and small size groups (80.86 ± 4.54 in the large group and 34.38 ± 1.67 in the small group). DeepLabV3+ could not detect calculi in the small size group. Another study classified kidney calculi into five different size groups results that were consistent with the above. The AUC was highest in the largest calculi group (>125 mm^3^) and decreased as the size decreased. The size of the kidney calculi included in the current study varied, but the majority of the calculi were small (<3 mm), which meets the criteria of the small size group in human medicine. Compared to the results of previous models in human medicine, our present models showed promising performance in detecting small calculi. Further studies, including more data on larger kidney calculi, can help improve the performance of the models.

In this study, the age of dogs with kidney calculi was significantly higher than that of dogs without calculi, which is consistent with the results of previous studies. In a human medicine study, it was reported that the prevalence of kidney calculi increased with age, and increasing age was considered a risk factor (38). However, a limitation of the present study is that we were not able to investigate and compare the ages at which calculi first developed, as the relevant data were not available due to the retrospective nature of the study. For consistency, the age at which the CT scan was obtained was considered to be the age of the dog, even if the dog had visited multiple times. Therefore, the age used in this study may have been biased toward older age.

The BW of the dogs with calculi was significantly lower than that of the dogs without calculi. Similarly, a recent study that investigated the prevalence and predictors of upper urinary tract uroliths in dogs found that dogs with upper urinary tract uroliths were significantly older and smaller than those without urolithiasis, which is consistent with our results (1). Also, a previous study showed that body height was inversely associated with the prevalence of kidney calculi diseases in human (39). Several factors have been considered as possible reasons for these results. If BW correlates directly with the ureteral diameter or length, the calculi are likely to spontaneously pass through the ureter more easily in those with a higher BW; however, studies on this topic are lacking. In addition, BW differs by breed and genetic factors might impact urolithiasis risk (1).

One of the limitations of this study was the relatively small size of the kidneys and kidney calculi in dogs compared to those in humans, which acted as an impediment factor for model development. Moreover, some calculi were smaller than the minimum pixel of the labeling program. Therefore, the margins of several calculi in the labeled image did not meet the actual margins of the calculi, which could have resulted the lower DSC values for small calculi in this study and could be considered as false negative. Another limitation of this study was the small number of CT scans included. Further studies with more CT scans may result in a better model performance. In addition, despite the result of this study, the lack of external validation in this study can be considered as a limitation. External validation using independent datasets from clinical fields with different image conditions and qualities would help demonstrate the applicability of the models in the context of practical approach. Another limitation of this study is that the data used for the model development were retrospectively collected from multicenter with different CT scanners, which acted as a major impediment factor of this study. Further prospective study with controlled data could result in the development of models with advanced performance.

In conclusion, the deep learning models proposed in this study showed promising results for the detection of small kidney calculi and highly encouraging results for the automatic segmentation of kidneys from pre-contrast CT images in dogs. These models can potentially assist clinicians in the detection of kidney calculi. Further studies using models that can automatically provide accurate volumes of calculi may have considerable clinical utility.

## Data availability statement

The original contributions presented in the study are included in the article/supplementary material, further inquiries can be directed to the corresponding author.

## Ethics statement

The animal studies were approved by the Institutional Animal Care and Use Committee of Jeonbuk National University. The studies were conducted in accordance with the local legislation and institutional requirements. Written informed consent was obtained from the owners for the participation of their animals in this study.

## Author contributions

YJ and HY: conception, design, drafting, and acquisition of data. YJ, GH, SL, KL, and HY: analysis, interpretation of data, revision for intellectual content, and final approval of the completed article. All authors contributed to the article and approved the submitted version.
